# Structural and Evolutionary Insights Into the Binding of Host Receptors by the Rabies Virus Glycoprotein

**DOI:** 10.3389/fcimb.2021.736114

**Published:** 2021-10-11

**Authors:** Manar E. Khalifa, Leonie Unterholzner, Muhammad Munir

**Affiliations:** ^1^ Division of Biomedical and Life Sciences, Faculty of Health and Medicine, Lancaster University, Lancaster, United Kingdom; ^2^ Department of Foot and Mouth Disease, Veterinary Serum and Vaccine Research Institute, Cairo, Egypt

**Keywords:** rabies virus, receptors, spillover, phylogenetic analysis, *in silico*, docking

## Abstract

Rabies represents a typical model for spillover of zoonotic viral diseases among multiple hosts. Understanding the success of rabies virus (RV) in switching hosts requires the analysis of viral evolution and host interactions. In this study, we have investigated the structural and sequence analysis of host receptors among different RV susceptible host species. Our extensive bioinformatic analysis revealed the absence of the integrin plexin domain in the integrin β1 (ITGB1) receptor of the black fruit bats in the current annotation of the genome. Interestingly, the nicotinic acetyl choline receptor (nAChR) interaction site with the glycoprotein (G) of RV was conserved among different species. To study the interaction dynamics between RV-G protein and the RV receptors, we constructed and analyzed structures of RV receptors and G proteins using homology modeling. The molecular docking of protein-protein interaction between RV-G protein and different host receptors highlighted the variability of interacting residues between RV receptors of different species. These *in silico* structural analysis and interaction mapping of viral protein and host receptors establish the foundation to understand complex entry mechanisms of RV entry, which may facilitate the understanding of receptor mediated spillover events in RV infections and guide the development of novel vaccines to contain the infection.

## Introduction

Rabies is a lethal zoonotic viral disease which causes serious behavioral changes and neurological disorders in a wide range of hosts with a high fatality rate of up to 100% (Hueffer et al., 2017). Rabies virus (RV) is an enveloped negative-stranded RNA virus and belongs to the family *Rhabdoviridae* with bullet-shaped virion particles with a size of ~200 nm. The viral genome encodes five transcriptional units for nucleocapsid protein (N), phosphoprotein (P), matrix protein (M), glycoprotein (G), and RNA-dependent RNA polymerase or large protein (L) (Jackson, 2013). Viral RNA is encapsulated by N protein which forms the ribonucleoprotein (RNP) and acts as a template for viral replication and transcription. The RNP together with P and L form the viral replication complex, which is surrounded by a lipid bilayer containing the viral G protein protruding as spikes from the viral surface. The M protein has been proposed to bridge the RNP and the cytoplasmic domain (CD) of G protein to form the bullet-shaped virion (Pulmanausahakul et al., 2008).

Owing to the location of G protein on the viral surface, it is considered the major determinant of tissue tropism. The RV exhibits a broad host spectrum, highlighting the importance of G protein in interacting with multiple host receptors (Jackson, 2013). The RV-G protein is a type I membrane glycoprotein which is translated on membrane-bound ribosomes and inserted cotranslationally into the endoplasmic reticulum (ER) in an unfolded form. The folding of transmembrane proteins occurs in three topologically and biochemically distinct environments: the ER lumen, the ER membrane, and the cytosol. The structural organization of RV-G protein indicated the constitution of three domains; ectodomain, transmembrane domain (TMD), and the cytoplasmic domain which can fold independently of each other (Maillard and Gaudin, 2002). Three different states have been demonstrated for the G protein. The native state (referred as “n”) is detected at the virus surface and is known to be responsible for receptor binding. The activated hydrophobic state (A) interacts with the target membrane as the primary step in the fusion process, and the fusion-inactive conformation state (I) (Gaudin et al., 1999). These distinct states are governed by pH equilibrium, where the I state is triggered by low pH, forming a more elongated conformation than that in the n state, rendering them antigenically different (Gaudin et al., 1999). Cleavage of the signal peptide results in the formation of the mature protein. The G protein undergoes cellular modification processes, whereby carbohydrates (glycans) are attached to specific amino acid side chains in protein. Appropriate folding of G glycoprotein is mainly dependent on the *N*-glycosylation sites, which increase the solubility of folding intermediates and facilitate the interaction with chaperones (Wojczyk et al., 2005).

Cellular receptors are regarded as the primary pathway through which viruses can gain access to the host. The ways by which viruses can unlock the host cells using the viral attachment proteins is considered the most fundamental aspect in viral invasion of the host cells. The RV interacts with a wide range of receptors by which it enters cells of different host species. Successful infection occurs only when a receptor can initiate the full viral life cycle from cell recognition to release the genome into the cell for replication and protein synthesis. Elucidating the preferences of RV in binding and attaching to certain types of host receptors is a point of interest which may provide insights into how entry mechanisms may be targeted therapeutically (Maginnis, 2018). Following attachment of the RV-G protein to host receptors, the virus is internalized when it reaches the endocytic zones. RV is transported into clathrin-coated pits by the filopodia. The filopodia are actin-enriched cell surface protrusions with which the cell probes the extracellular environment (Xu et al., 2015).

Nicotinic acetyl choline receptor (nAChR), a well-known host receptor for RV, is a pentameric ligand-gated ion channel which is present in neuromuscular junctions, mediating intraneuronal communication in central and peripheral nervous systems (Lafon, 2005). Recently, it has been observed that cells lacking nAChR are still susceptible to RV infection. Additionally, RNA interference (RNAi)-mediated depletion of different receptors was carried out to explore the role of the various RV receptors. The most recently discovered RV receptor is integrin beta 1 (ITGB1) (Shuai et al., 2019). ITGB1 is a transmembrane cell surface receptor and consists of one α and one β subunits. The expression of ITGB1 is predominantly observed in the skeletal muscle. The cell lines expressing ITGB1 are human embryonic kidney cells (HEK293) and mouse neuroblastoma cells (N2a) (Shuai et al., 2019). Another novel RV receptor, metabotropic glutamate receptor subtype 2 (mGluR2), has recently been identified. It belongs to the G protein-coupled receptor family that is abundant in the central nervous system. Several human and murine cell lines (i.e., HEK293, N2a and neuroblastoma cells SK-N-SH, SK cells) express mGluR2 receptors and can be successfully infected with RV (Wang et al., 2018). Neural cell adhesion molecule (NCAM) is another well-known RV receptor which is a cell adhesion glycoprotein of the immunoglobulin superfamily, and it is mainly concentrated in synaptic regions and at the neuromuscular junction (NMJs). Its main role is mobilization and cycling of synaptic vesicles in addition to synaptogenesis (Lafon, 2005). Additionally, other components of the cell membrane, such as gangliosides and heparin sulfate were recognized to play a role in the entry of RV to hosts (Lafon, 2005).

Despite the availability of substantial information on the RV infectivity, there is a gap in understanding the mechanism by which the G protein can interact with different receptors for initiation of the infection. In the current study, we aim to underpin the evolutionary differences among different RV receptors in humans, dogs, and bats with disclosure of the possible *in silico*-predicted mechanisms by which the G protein can interact with the different receptors in different host species. The presented data provide insights into these interactions to open avenues for the prevention and control of RV infection in future.

## Materials and Methods

### Construction of Data Sets

To investigate the differences among RV receptors in different species, protein sequences were retrieved from NCBI (Agarwala et al., 2018) by BLAST search. All sequences for each of the proteins were downloaded and collated in a FASTA format.

### Alignment of the Protein Sequences

Multiple sequence alignment for protein sequences of host receptors were performed using DNASTAR Laser Gene version 17.0.2.1 (Burland, 2000), using the MUSCLE method (Edgar, 2004).

### Primary Structure Analysis of Receptors

The protein sequences of ITGB1, mGluR2, nAChR, and NCAM receptors in different host species (humans, dogs, and black fruit bats) were retrieved from GenBank (Agarwala et al., 2018). Physicochemical properties of proteins, molecular weight, theoretical isoelectric point (pI), instability index, and grand average of hydropathicity (GRAVY) were identified by ProtParam (Gasteiger et al., 2005).

### Domain Organization for RV Receptors

Domain organization for different RV receptors in different species were analyzed by Pfam (http://pfam.xfam.org) (Finn et al., 2016), InterPro (http://www.ebi.ac.uk/interpro/) (Mitchell et al., 2015), and CDD databases (Marchler-Bauer et al., 2015). Schematic diagrams for domains in the different protein receptors have been represented by PROTTER software (Omasits et al., 2014). Signal peptide, transmembrane, and low complexity regions within receptors in different species were retrieved from SMART (Letunic et al., 2015) and SignalP databases (Armenteros et al., 2019).

### 3D Structure Model Building and Quality Assessment

The 3D structure models for RV receptors (ITGB1, mGluR2, nAChR, and NCAM proteins), RV-G protein of Egyptian strain (QEU57979.1), and RV-G protein of bat-related group (BAE95290.2) were generated using I-TASSER (Yang et al., 2010). The 3D structures generated by I-TASSER were based upon threading, fragment assembly, and iteration. The best model was selected according to the confidence score (C-score) which represented the quality of predicted models by I-TASSER. The C-score range was between (−5 and −2), where the higher the C-score, the higher the confidence of a model and vice versa. After predicting the protein model, structure and stereochemical analyses were performed and the predicted 3D structures were visualized and annotated using PyMOL software (DeLano, 2002).

### Molecular Docking Simulations

The predicted structures were used for protein-protein docking studies using GRAMM-X software (Tovchigrechko and Vakser, 2006). Docking studies were performed for the Egyptian RV-G protein against each of RV receptors from human, dog, and black fruit bat. In addition, *in silico* interactions between RV-G protein related to bat strain were mapped with different receptors.

### Analysis of the Docking Complex

The docking complexes obtained from GRAMM-X were uploaded to PDBsum (Laskowski et al., 2018) and PDBePISA (Battle, 2016) servers for analysis of the protein-protein interactions. Identification of hydrogen bonds, interacting interfaces, nonbonded contacts, salt bridges, Gibb’ free energy of binding (ΔG^int^, kcal/mol), pores, and tunnels in protein complexes were carried out. Mapping of the docking complexes was performed using PYMOL software (DeLano, 2002).

## Results

### Computational Analysis of Primary Structure of RV Receptors

The physicochemical properties of ITGB1, mGluR2, nAChR, and NCAM were compared among different species (human, dog, and black fruit bat) based on their amino acid (a.a.) composition as summarized in **Table 1**. Investigation of the hydrophobic nature of the RV receptors was assessed by GRAVY values. The range of GRAVY values for ITGB1 and NCAM were negative, revealing their overall hydrophilic nature. In contrast, positive GRAVY values identified in mGluR2 and nAChR proteins indicated their hydrophobic nature. To test the stability of different receptors, the instability index values were estimated. Instability indices for mGluR2, nAChR, and NCAM were all below 40, indicating the stability of these proteins in all species examined. However, the ITGB1 in human and dogs were considered relatively unstable, compared with ITGB1 from black fruit bat which had an instability index below 40. The number of amino acid residues, signal peptide, transmembrane, and low complexity regions were predicted and are summarized in **Table 1**. Interestingly, the signal peptide in black fruit bat ITGB1 was missing according to SMART and SignalP databases in the sequence that is available in the current genomic annotation.

**Table 1:** Physicochemical parameters computed using expasy’s protparam tool for signal peptide, transmembrane, and low complexity regions of RV receptors were determined by SMART.

	ITGB1			mGluR2			nAChR			NCAM		
	Human	Dog	Bat	Human	Dog	Bat	Human	Dog	Bat	Human	Dog	Bat
Number of a.a.	798	801	741	872	872	872	482	457	457	858	857	760
Molecular weight	88,415	88,592	82,000	95,567	95,714	95,579	54,545	51,878	51,911	94,574	94,491	83,788
Theoretical pI	5.27	5.33	5.24	8.50	8.50	8.49	5.78	5.56	5.82	4.79	4.82	4.80
Instability index	41.12	40.7	39.2	37.3	39.32	36.76	31.9	35.5	31.9	36.6	36.6	39.4
GRAVY	−0.40	−0.39	−0.42	0.09	0.09	0.10	0.18	0.21	0.20	−0.41	−0.40	−0.31
Signal peptide (aa)	1–20	1–20	–	1–18	1–18	1–18	1–20	1–20	1–20	1–19	1–19	1–19
Transmembrane region (a.a)	729–751	729–751	672–694	4–13	4–16	4–16	258–280	233–255	233–255	724–746	723–745	–
				301–314	483–498	301–314	292–309	267–284	267–284			
				795–809	795–809	795–809	319–341	294–316	294–316			
				833–854	829–853		454–476	429–451	429–451			
Low complexity regions (a.a)	729–757	729–757	672–700	–	–	–	6–19	6–12	5–20	252–261	723–738	596–615
							289–306	264–281	267–284	807–819	808–818	746–759
							328–341	303–316	294–316			
									429–451			

The accession number of ITGB1 in human, dog, and bat are NP_596867.1, XP_022261846.1, and XP_006903905.2, respectively. The mGluR2 accession numbers in human, dog, and bat are NP_000830.2, XP_541867.2, and XP_006909212.1, respectively. The nAChR accession numbers are NP_001034612.1, NP_001003144.1, and XP_006921280.1 in human, dog, and bat, respectively. The NCAM accession numbers used in this study are NP_851996.2, XP_005619557.1, and XP_006912868.1, respectively.

### Domain Organization of Different Receptors

**Figure 1:**
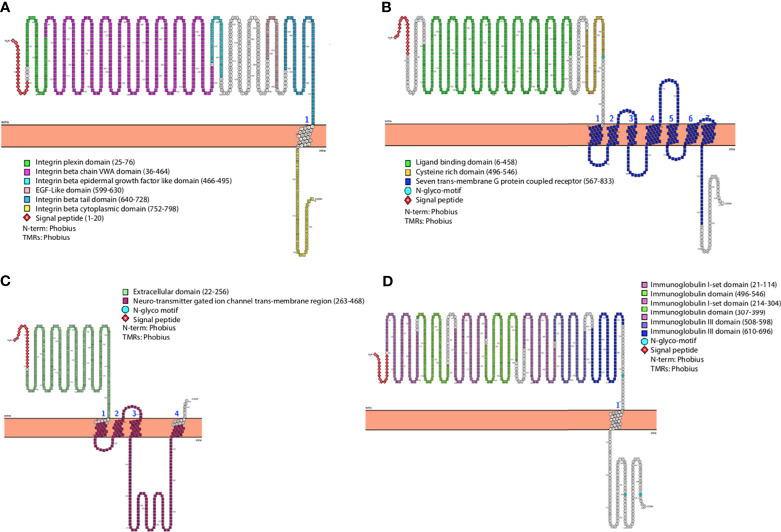
General representation of human **(A)** ITGB1, **(B)** mGluR2, **(C)** nAChR, and **(D)** NCAM highlighting different domains and most relevant features. The graphs were generated using PROTTER v1.0.

A schematic representation of the domain organization of human ITGB1, mGluR2, nAChR, and NCAM receptors are shown in **Figures 1A**–**D**, respectively. The 3D structures of the receptors highlighting each domain are also shown (**Supplementary Figures S1A**–**D**). Analysis of the human ITGB1 a.a. sequence (**Figure 1A**) revealed six distinct domains. The integrin plexin domain, a short disulfide-rich domain (a.a. from 25 to 76) located at the N-terminus of integrin beta chains, was also present in dog ITGB1, but interestingly was the only domain absent in black fruit bat (**Supplementary Figure S1A**). The longest domain within ITGB1 is the von Willebrand A (VWA) domain, encompassing the region from a.a. 36 to 464. The smallest domain in ITGB1 is the integrin beta epidermal growth-like factor domain 1 (a.a. 466–495). In addition, an epidermal growth factor (EGF)-like domain 2 (a.a. 599–630) and integrin beta tail domain (a.a. 640–728) were also identified. The most distal domain was the cytoplasmic domain (a.a. 752–798). The mGluR2 receptor was structurally divided into three domains (**Figure 1B**). The large extracellular region is the ligand binding domain of the group II metabotropic glutamate receptor (a.a. 6–458). The cysteine-rich domain (CRD) (a.a. 469–546) is characterized by highly conserved cysteine residues forming disulfide bridges. Linked to the CRD domain is a transmembrane domain composed of seven transmembrane helices (7 TMD) (a.a. 567–833 a.a.). All domains were similarly present in dogs and black fruit bat. The domain organization analysis of the nicotinic acetyl-choline receptor nAChR (**Figure 1C**) demonstrated a large conserved extracellular domain (a.a. 22–256). Four transmembrane regions named as neurotransmitter gated ion channel (a.a. 263–468) were followed by a cytoplasmic loop (Albuquerque et al., 2009). A similar domain arrangement was also present in dog and black fruit bat within the nAChR. Analysis of the neural cell adhesion molecule (NCAM) domain architecture revealed an extracellular portion of NCAM which is composed of five N-terminal Ig-like domains and two fibronectin type III domains which form a dimeric glycoprotein composed of disulfide-linked subunits. NCAM domain arrangement was the same in dog and black fruit bat (**Figure 1D**).

### Protein Sequence Alignment

**Figure 2:**
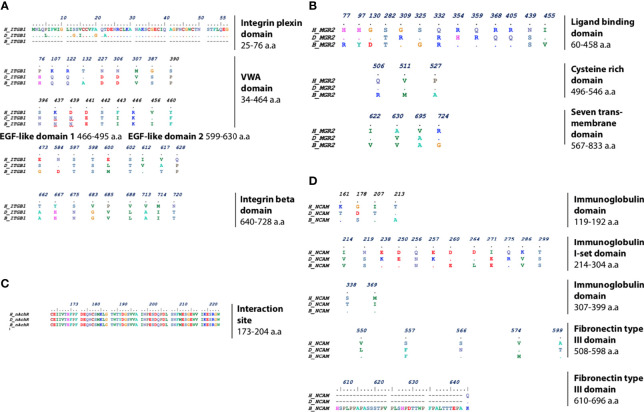
Multiple protein sequence alignment of **(A)** ITGB1, highlighting the different residues in integrin plexin domain, VWA domain, EGF-like domain (1 and 2), and integrin beta tail domain. **(B)** mGluR2, highlighting the different residues in ligand-binding domain, cysteine-rich domain, and seven transmembrane domains. **(C)** nAChR, highlighting the nAChR interaction site with RV-G among human, dog, and black fruit bat. **(D)** NCAM, highlighting the different residues in immunoglobulin and fibronectin domains between human, dog, and black fruit bat domains.

Analysis of sequence similarities of receptors among different species was crucial for mapping the differences within the interaction site of the receptors with the RV-G protein. For each of the four RV receptors, protein sequences from human, dog, and black fruit bat were aligned (**Figures 2A**–**D**). Analysis of the ITGB1 protein sequences from human, dog, and black fruit bat (**Figure 2A**) revealed that the VWA domain represented the most variable region among species, where dog showed 15 a.a. differences in comparison with human and 18 a.a were variable between black fruit bat and human. The area of greatest homology among the ITGB1 protein was the EGF-1 domain where only one a.a. varied between species. There were five different a.a. residues in the integrin plexin domain between human and dog. Similarly, within the EGF-2 domain, four and five a.a. residues showed differences in black fruit bat and dog, compared with human. Both dog and black fruit bat showed nine a.a. residue difference in the tail domain relative to human. The differences in these residues (1–728 a.a) may be crucial, since the ITGB1 ectodomain constitutes the interaction site with the RV-G protein, as was pointed out in a previous study (Shuai et al., 2019). Our results highlight the sequence homology within the mGluR2 protein alignment among the different species (**Figure 2B**). The ligand-binding domain showed the least sequence similarity between species where eight and 10 a.a. residues were different in black fruit bat and dog, respectively, in comparison with human. The CRD domain only showed three a.a. differences between black fruit bat, dog, and human. Only four amino acid residues were different in the transmembrane domain between human, dog, and black fruit bat. Considering that the binding site of RV-G with nAChR was previously mapped to be on the αx subunit between residues 173 and 204 (Lafon, 2005), one major finding in our study (**Figure 2C**) was the high conservation of this region among human, dog, and black fruit bat. The interaction site of NCAM with RV-G protein has not been previously determined. Our results showed the a.a. differences of NCAM domains among the different species. As shown in **Figure 2D**, the first immunoglobulin domain and second and third immunoglobulin I-set domains were the most conserved regions among all species. On the other hand, the first immunoglobulin I-set domain showed the most variable region among the three species where eight and 10 a.a. residues differ in black fruit bat and dog, respectively, compared with human. Black fruit bat NCAM displayed the highest variability in its fibronectin type III domain where 26 residues were variable, while only one residue differed in dog with respect to human.

### Analysis of the Predicted 3D Structures of Receptors

The 3D structures of different receptors were predicted using the ITASSER online server. The best predicted model structures were chosen according to the maximum confidence score which was calculated according to threading templates significance (**Supplementary Figures S1A**–**D**). The C-score ranged from −5 to −2 (the higher the value, the higher the confidence and vice versa). For human ITGB1, a model with a C-score of 0.40 was selected; for dog ITGB1, a model with a C-score of 0.37 was chosen; and for bat, a model with C-score of 0.23 was selected. For mGluR2, 3D structures of the dog (C-score, −0.13) human (C-score, −0.13), and black fruit bat (C-score, −0.19) were chosen. For nAChR, the best model in human had a C-score of −0.37, dog with a C-score of 0.05, and black fruit bat with a C-score of 0.35. NCAM C-scores of 3D structures were as follows: human, −0.69; dog, −0.79; and black fruit bat, 0.10.

### Protein-Protein Interaction Prediction

**Figure 3:**
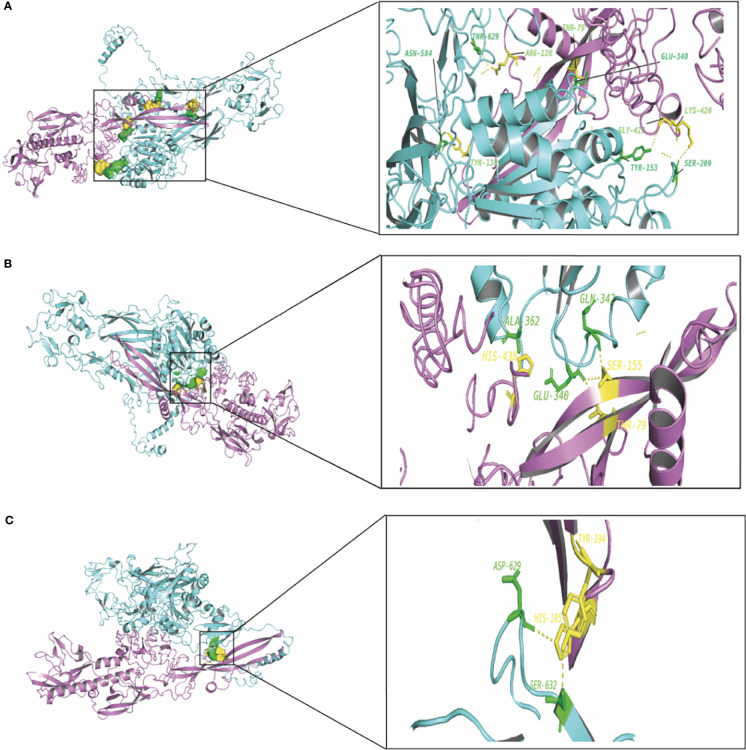
**(A)** Residues involved in hydrogen bonds within the docking complex of human ITGB1 with RV-G protein, Egyptian strain (QEU57979.1). **(B)** Residues involved in hydrogen bonds within the docking complex dog ITGB1-RV-G protein, Egyptian strain (QEU57979.1). **(C)** Residues involved in hydrogen bonds within the docking complex black fruit bat ITGB1-RV-G protein, Egyptian strain (QEU57979.1). Docking complex (ITGB1-RV-G Egyptian strain); ITGB1 colored in cyan, interacting a.a residues colored in green, RV-G protein colored in violet, interacting a.a residues colored in yellow.

**Figure 4:**
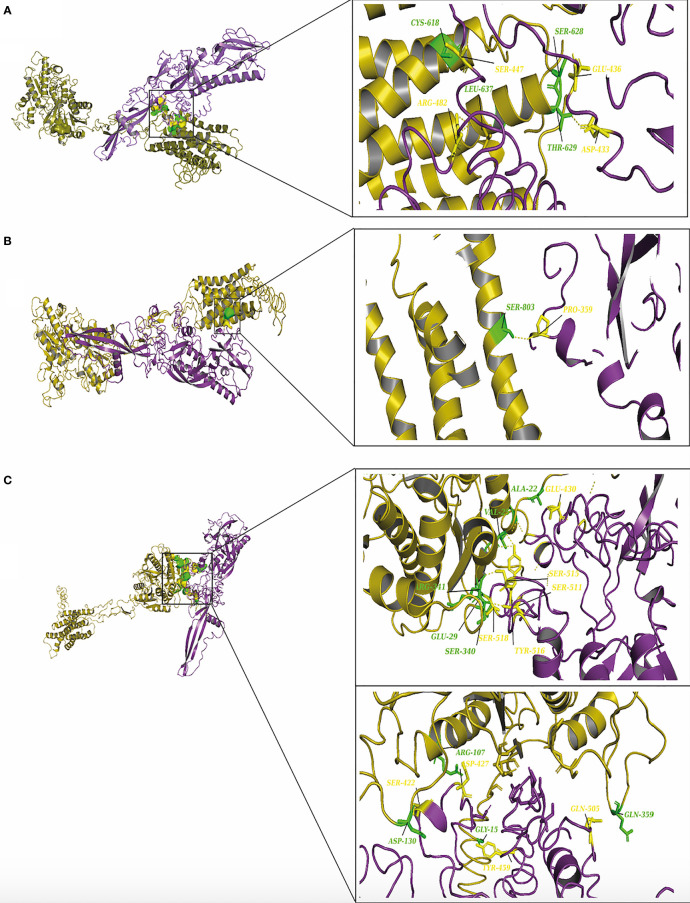
**(A)** Residues involved in hydrogen bonds within the docking complex human mGluR2-RV-G protein, Egyptian strain (QEU57979.1). **(B)** Residues involved in hydrogen bonds within the docking complex dog mGluR2-RV-G protein, Egyptian strain (QEU57979.1). **(C)** Residues involved in hydrogen bonds within the docking complex black fruit bat mGluR2-RV-G protein, Egyptian strain (QEU57979.1). Docking complex (mGluR2-RV-G protein, Egyptian strain); mGluR2 colored in golden yellow, interacting a.a residues colored in green, RV-G protein colored in violet, interacting a.a residues colored in yellow.

**Figure 5:**
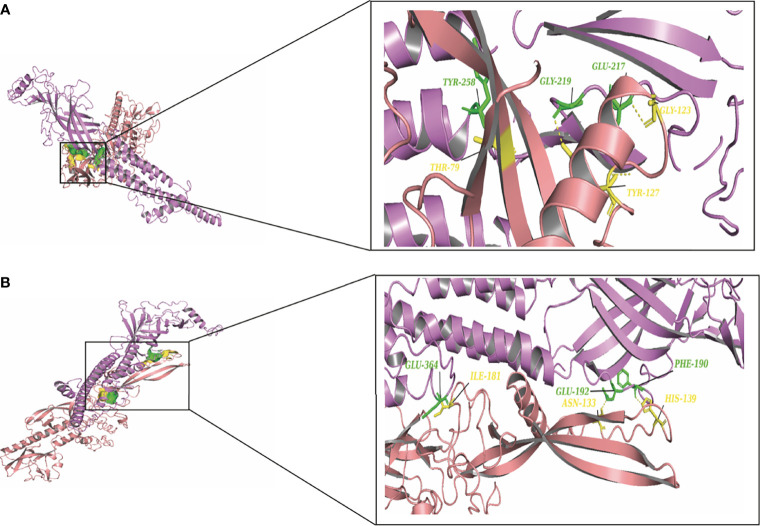
**(A)** Residues involved in hydrogen bonds within the docking complex human nAChR-RV-G Egyptian strain (QEU57979.1). **(B)** Residues involved in hydrogen bonds within the docking complex black fruit bat nAChR RV-G Egyptian strain (QEU57979.1). Docking complex (nAChR-RV-G Egyptian strain); nAChR colored in magenta, interacting a.a residues colored in green, RV-G protein colored in salmon, interacting a.a residues colored in yellow.

**Figure 6:**
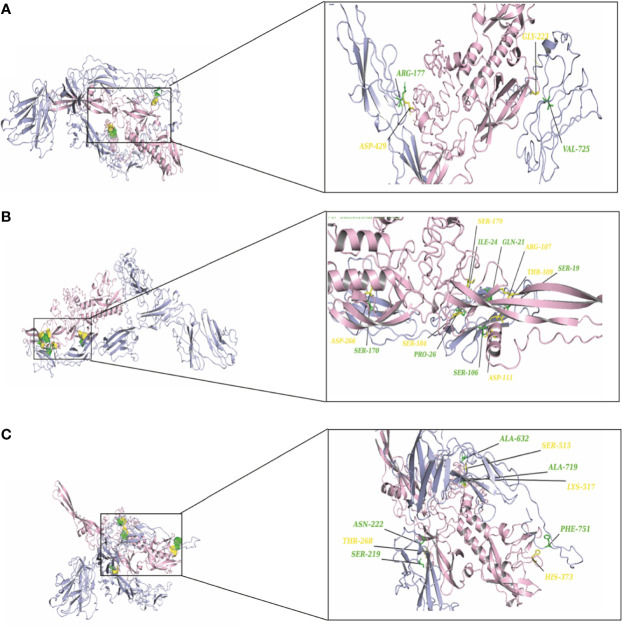
**(A)** Residues involved in hydrogen bonds within the docking complex human NCAM-RV-G Egyptian strain (QEU57979.1). **(B)** Residues involved in hydrogen bonds within the docking complex dog NCAM-RV-G Egyptian strain (QEU57979.1). **(C)** Residues involved in hydrogen bonds within the docking complex black fruit bat NCAM-RV-G Egyptian strain (QEU57979.1). Docking complex (NCAM-RV-G Egyptian strain); mGluR2 colored in light blue, interacting a.a residues colored in green, RV-G protein colored in light pink, interacting a.a residues colored in yellow.

To elucidate the mechanism by which the RV-G protein interacts with different receptors, protein-protein docking was performed using GRAMMX. Analysis of the docking complexes was resolved through PDBsum and PDBePISA servers. Additionally, mapping the interacting hydrogen bonds, salt bridges, and the ΔG^int^ was also performed (**Table 2**). The ΔG^int^ value which expresses the solvation free energy gain upon assembly formation (total solvation energies of assembled structures-solvation energies of isolated structures) was determined as well (Pantsar and Poso, 2018). The RV-modeled 3D structure of Egyptian strain G protein was utilized to undertake the docking against different receptors. The docking complex of human ITGB1 and Egyptian RV-G protein indicated five interactions mediated by hydrogen bonds between residues of ITGB1 VWA and EGF-1-like domain with the RV-G protein in addition to formation of one salt bridge between Glu^340^ of human ITGB1 and His^438^ of the RV-G protein (**Figure 3A**). A relatively more stable docking complex between dog ITGB1 VWA domain and RV-G protein of ΔG^int^ −20.8 kcal/mol was mapped (**Figure 3B**). The stability of the docking complex might be due to formation of four hydrogen bonds between VWA domain of dog ITGB1. Four salt bridges between Lus^156^, Asp^287^, Glu^340^, and Glu^347^ in dog ITGB1 and Asp^429^, His^105^, His^438^, and Arg^103^ in the RV-G protein were identified. Predicted interactions of the integrin beta tail domain from black fruit bat ITGB1 showed bonding with RV-G protein through three hydrogen bonds (**Figure 3C**). Our results showed that the G protein ectodomain is responsible for binding to ITGB1 in different hosts (**Figures 3A**–**C**). Our modeling of the interaction between mGluR2 in human and dog with RV-G protein showed that the interactions were only mediated by the hydrogen bonds in the seven-transmembrane domain of mGluR2 (**Figures 4A, B**). Intriguingly, 10 hydrogen bonds were at the interface between the ligand-binding domain of mGluR2 from black fruit bat and the RV-G protein (**Figure 4C**), along with formation of four salt bridges between Lys^24^, Arg^107^, and His^129^ in black fruit bat mGluR2 and Glu^430^, Asp^427^, and Asp^420^ in the RV-G protein. Interestingly, the G protein ectodomain, transmembrane, and cytoplasmic domains all appear to play a role in interactions of G protein with mGluR2 in different hosts (**Figures 4A**–**C**). The nAChR extracellular domain of human and black fruit bat interacted with RV-G protein through three hydrogen bonds (**Figures 5A, B**). Surprisingly, the docking complex with dog nAChR showed neither hydrogen bonds nor any salt bridges with the RV-G protein which needs further investigation. It was observed that the G protein ectodomain was responsible for interactions with nAChR in human and bat (**Figures 5A, B**). Two hydrogen bonds mediate the interaction between human NCAM and RV-G in the docking complex (**Figure 6A**). A salt bridge was noticed between residues Arg^177^ in human NCAM and Asp^429^ within the RV-G protein. Modeling of the dog NCAM-RV-G docking complex demonstrated nine hydrogen bonds (**Figure 6B**). A total of five hydrogen bonds were mapped in the docking complex between black fruit bat NCAM and RV-G protein (**Figure 6C**). The RV-G ectodomain was the interacting part with human and dog NCAM, while both the RV-G ectodomain and cytoplasmic domains may interact with bat NCAM.

**Table 2:** Analysis of docking complex ITGB1-RV-G, mGluR2-RV-G, nAchR-RV-G, and NCAM-RV-G proteins in human, dog, and black fruit bat, highlighting the hydrogen bonds, interacting interfaces, nonbonded contacts, salt bridges, ΔG^int^, pores, and tunnels within the docking complex.

Docking-complex	Hydrogen bonds	Interacting interfaces	Non bonded contacts	Salt bridges	ΔG^int^ (kcal/mol)	Pores	Tunnels
ITGB1	5	39	233	1	−21.5	24	2
Human		36					
ITGB1	4	42	236	4	−20.8	13	2
Dog		38					
ITGB1	3	35	294	−	−26.2	13	9
Black fruit bat		33					
mGluR2	4	32	206	−	−29.7	9	3
Human		31					
mGluR2	1	37	278	−	−32.6	12	8
Dog		38					
mGluR2	10	33	224	4	−18.4	12	1
Black fruit bat		33					
nAChR	3	27	207	−	−22.8	10	10
Human		28					
nAChR	−	34	219	−	−31.9	9	8
Dog		37					
nAChR	3	24	188	−	−24.7	6	9
Black fruit bat		30					
NCAM	2	27	187	1	−14.9	23	10
Human		28					
NCAM	9	36	280	−	−20.9	16	8
Dog		34					
NCAM	5	37	194	−	−19.3	11	10
Black fruit bat		31					

**Figure 7:**
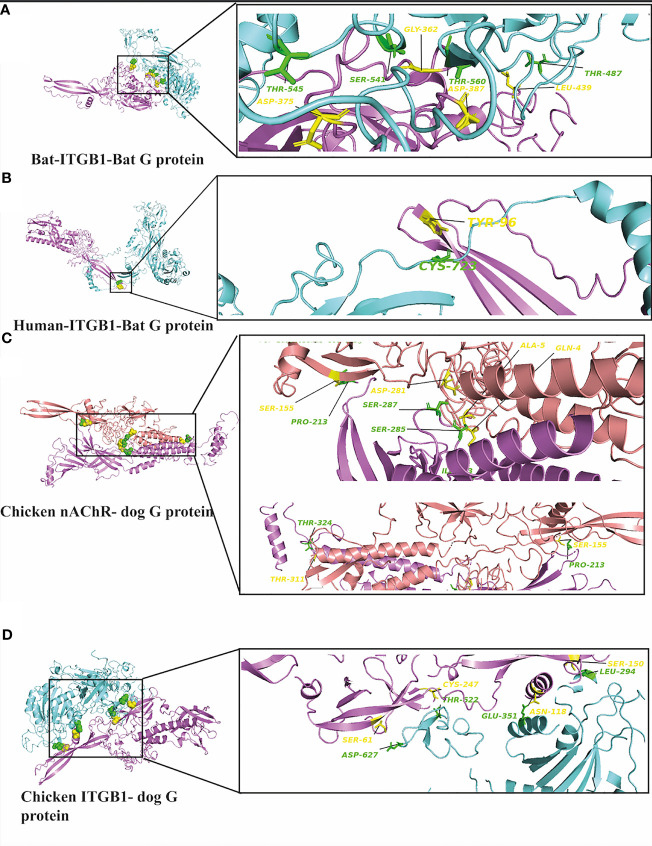
**(A)** Residues involved in hydrogen bonds within the docking complex bat ITGB1-bat RV-G protein (BAE95290.2). **(B)** Residues involved in hydrogen bonds within the docking complex human ITGB1-bat RV-G protein (BAE95290.2). **(C)** Residues involved in hydrogen bonds within the docking complex chicken nAChR with RV-G Egyptian strain protein (QEU57979.1). **(D)** Residues involved in hydrogen bonds within the docking complex chicken ITGB1 with RV-G Egyptian strain (QEU57979.1). ITGB1 colored in cyan, a.a residues colored in green, RV-G protein colored in violet, a.a residues colored in yellow, nAChR colored in magenta, a.a residues colored in green, RV-G protein colored in salmon, a.a residues colored in yellow.

Interestingly, we have performed further docking analysis and compared the interaction of bat ITGB1 with the G protein of the bat RV group as shown in **Figure 7**. Our results show the formation of four hydrogen bonds between bat ITGB1 and bat-related RV-G protein (**Figure 7A**), while only two hydrogen bonds were mapped between bat ITGB1 and the G protein of the dog-related group (**Figure 3C**). To elucidate if the interaction of human ITGB1 will be stronger with bat- or dog-related RV-G proteins, we modeled the docking complex of human ITGB1 with bat RV-G protein (**Figure 7B**). The best model predicted an interaction mediated by only one hydrogen bond between human ITGB1 and bat RV-G protein, in contrast to the five hydrogen bonds created upon interaction of human ITGB1 with dog RV-G protein (**Figure 3A**). For checking the accuracy of the docking results, we have tested if nonsusceptible host to RV (chicken) will show any interacting residues with G protein. Two docking complexes were modeled as follows: chicken nAChR–dog G protein and chicken ITGB1–dog G protein. Unexpectedly, chicken nAChR–dog G protein complexes showed seven hydrogen bonds (**Figure 7C**). While in chicken ITGB1–dog G protein, four hydrogen bonds were mapped (**Figure 7D**).

## Discussion

Little is known about the mechanisms by which RV crosses species barriers. Studying the differences among RV receptors in different species through which the RV is capable to jump among different host species will provide novel insights into controlling spillover events. The capability of RV-G protein to bind wide range of receptors from different protein families and expressed by diverse cell types remain elusive and puzzling (Lafon, 2005; Wang et al., 2018; Shuai et al., 2019). In order to unravel some of these mechanisms, we performed structural and protein-protein interaction analysis of all known RV receptors and investigated the possible mechanisms by which the G protein may enter into diverse cell types in different host species. Interestingly, some differences were observed especially for the ITGB1 domain organization of black fruit bat which showed absence of integrin plexin domain and signal peptide (**Table 1**). Signal peptides have an important role in protein sorting and localization and its absence is a matter of interest and warrant future investigation. The absence of an N-terminal signal peptide among orthologous proteins might be linked to the absence of the integrin plexin domain (N-terminal domain) in ITGB1 from black fruit bat in comparison with ITGB1 in other species (Hönigschmid et al., 2018). On the molecular level, protein-protein interactions are the basis of life. Mapping and modeling the protein interactions through computational approaches (docking) can improve our understanding of the interactions occurring *in vivo*, though with less accuracy (Tovchigrechko et al., 2002). In the current study, we analyzed the protein-protein interactions between the RV-G protein and ITGB1 in different species (**Figures 3A**–**C**). Results obtained from ITGB1 and RV-G protein docking are consistent with previous studies which identified that the interaction site between ITGB1 and RV-G protein within residues 1–728 a.a on the ITGB1 ectodomain (Shuai et al., 2019). Also, our analysis showed that the G protein ectodomain was the interacting site with ITGB1 in human, dog, and bat. Regarding mGluR2, none of the previous studies have identified the interaction site with RV-G protein. Our analysis for the docking results indicated the importance of the transmembrane domain specifically in humans and dogs to interact with RV-G protein. In contrast, in black fruit bat mGluR2, the hydrogen bond with RV-G protein was within the ligand binding domain. The diversity in orthologous protein domains interacting with RV-G protein is plausible. Despite the sequence similarity shared by proteins, divergent functions and interactions are commonly observed (Hönigschmid et al., 2018). Although, the interaction site for nAChR with RV-G protein has been proposed to be within 173–204 a.a region (Lafon, 2005), our results predict different interacting a.a. residues, however within the same domain. These findings highlight the importance of future *in vitro* and *in vivo* studies to gain further molecular mechanistic insights. The interaction site of NCAM with the RV-G protein has not been determined in previous studies. Our results demonstrated that hydrogen bonds bonded mainly within immunoglobulin-like domains and fibronectin III-like domain which may define the interaction between the virus and cell receptor. Our focus in hydrogen bond mapping within interaction complexes was primarily due to their known roles in improving the stability of the interacting protein complexes (Nilofer et al., 2017). Differences of the binding a.a. residues between the two docking complexes: (bat ITGB1-bat RV-G) and (human ITGB1-bat RV-G) may suggest the involvement of the residues near the conserved a.a. in bat-related RV-G mentioned above (**Figure 3**) in binding to bat receptors. Since virus attachment to cellular receptors is considered only the preliminary step for viral infection, this might explain our prediction that the RV-G protein can bind to chicken receptors, even though chickens are not hosts of RV infection. Therefore, we can hypothesize that penetration or other steps involved in viral infection might be the barrier to infect chickens and other non-host species. In the current study, predicted protein-protein interactions were performed with Gramm-X software which is based on rigid body docking utilizing the Fast Fourier transform (FFT) algorithm (Tovchigrechko and Vakser, 2006). The FFT algorithm allows the determination of the best surface match between molecules based on shape complementarity (Tovchigrechko and Vakser, 2006). This method has clear limitations represented in reduced accuracy due to large conformational changes formed upon binding of protein complexes (Desta et al., 2020). In addition to the possibility of large movements during binding which ultimately may result in transient or weak binding (Pons et al., 2010). Moreover, the reliability of docking on structural models of proteins generated by computational analysis render them more prone to errors (Szilagyi and Zhang, 2014). Besides the mentioned limitations, Gramm-X software does not allow for the selection of specific glycosylation sites in modulating the interactions between RV-G and receptors. Thus, the stability of the generated docking complex might have been affected. Since, anticipating the glycosylated sites might have resulted in higher free energy which is ultimately known to affect the stability of docking complex (Shental-Bechor and Levy, 2008).

Taken together, our *in silico* analysis, unraveled some of the most crucial receptors utilized by RV for entry purposes. These analyses establish the foundations for future research to understand the preference and mutual importance of each of these receptors for the entry mechanisms of RV. Additionally, these structure-guided insights will establish a foundation on the host-specific differences that may help to understand the spillover of the RV among different hosts.

## Data Availability Statement

The datasets presented in this study can be found in online repositories. The names of the repository/repositories and accession number(s) can be found in the article/**Supplementary Material**.

## Author Contributions

MEK and MM contributed to the conceptualization of the study. MK contributed to formal analysis. MEK and MM contributed to validation. MEK performed *in vitro* experiments. MEK contributed to original draft preparation. MM and LU contributed to review and editing. MM and LU contributed to supervision. All authors contributed to the article and approved the submitted version.

## Funding

This study was funded by the Biotechnology and Biological Sciences Research Council (BBSRC) (BB/M008681/1 and BBS/E/I/00001852) and the British Council (172710323 and 332228521). This research was funded by the British Council. The Ph.D. studies of MEK have been financially supported by Newton Mosharafa-Fund (Bureau ID: NMM11/19) and the Egyptian Ministry of Higher Education and Scientific Research, Cultural Affairs and Mission Sector, Egypt.

## Conflict of Interest

The authors declare that the research was conducted in the absence of any commercial or financial relationships that could be construed as a potential conflict of interest.

## Publisher’s Note

All claims expressed in this article are solely those of the authors and do not necessarily represent those of their affiliated organizations, or those of the publisher, the editors and the reviewers. Any product that may be evaluated in this article, or claim that may be made by its manufacturer, is not guaranteed or endorsed by the publisher.

## References

[B1] Agarwala R. Barrett T. Beck J. Benson D. A. Bollin C. Bolton E.. (2018). Database Resources of the National Center for Biotechnology Information. Nucleic Acids Res. 46, D8–D13. doi: 10.1093/nar/gkx1095 29140470 PMC5753372

[B2] Albuquerque E. X.. (2009). Mammalian Nicotinic Acetylcholine Receptors: From Structure to Function. Physiol. Rev. 89, 73–120. doi: 10.1152/physrev.00015.2008.Mammalian 19126755 PMC2713585

[B3] Armenteros J. J. Tsirigos K. D. Sønderby C. K. Petersen T. N. Winther O. Brunak S.. (2019). SignalP 5.0 Improves Signal Peptide Predictions Using Deep Neural Networks. Nat. Biotechnol. 37, 420–423. doi: 10.1038/s41587-019-0036-z 30778233

[B4] Battle G. M. (2016) PDBePISA : Identifying and Interpreting the Likely Biological Assemblies of a Protein Structure. Available at: https://www.semanticscholar.org/paper/PDBePISA-%3A-Identifying-and-interpreting-the-likely-Battle/2154b7c24212f0c21793a008a7255d328d6d4b23.

[B5] Burland T. G. (2000). Sequence Analysis Using DNASTAR ‘ s Lasergene Software Suite. Methods Mol. Biol. 132, 71–91. doi: 10.1385/1-59259-192-2:71 10547832

[B6] DeLano W. L. (2002). Pymol: An Open-Source Molecular Graphics Tool. {CCP4} Newsl. Protein Crystallogr. 40, 1–8.

[B7] Desta I. T. Porter K. A. Xia B. Kozakov D. Vajda S. (2020). Performance and Its Limits in Rigid Body Protein-Protein Docking. Structure 28, 1071–1081.e3. doi: 10.1016/j.str.2020.06.006 32649857 PMC7484347

[B8] Edgar R. C. (2004). MUSCLE: Multiple Sequence Alignment With High Accuracy and High Throughput. Nucleic Acids Res. 32, 1792–1797. doi: 10.1093/nar/gkh340 15034147 PMC390337

[B9] Finn R. D. Coggill P. Eberhardt R. Y. Eddy S. R. Mistry J. Mitchell A. L.. (2016). The Pfam Protein Families Database: Towards a More Sustainable Future. Nucleic Acids Res. 44, D279–D285. doi: 10.1093/nar/gkv1344 26673716 PMC4702930

[B10] Gasteiger E. Hoogland C. Gattiker A. Duvaud S. Wilkins M. R. Appel R. D.. (2005). "The Proteomics Protocols Handbook," in Proteomics Protoc. Handb., edition 1. 571–608. doi: 10.1385/1592598900

[B11] Gaudin Y. Moreira S. Bénéjean J. Blondel D. Flamand A. Tuffereau C. (1999). Soluble Ectodomain of Rabies Virus Glycoprotein Expressed in Eukaryotic Cells Folds in a Monomeric Conformation That Is Antigenically Distinct From the Native State of the Complete, Membrane-Anchored Glycoprotein. J. Gen. Virol. 80, 1647–1656. doi: 10.1099/0022-1317-80-7-1647 10423132

[B12] Hönigschmid P. Bykova N. Schneider R. Ivankov D. Frishman D. (2018). Evolutionary Interplay Between Symbiotic Relationships and Patterns of Signal Peptide Gain and Loss. Genome Biol. Evol. 10, 928–938. doi: 10.1093/gbe/evy049 29608732 PMC5952966

[B13] Hueffer K. Khatri S. Rideout S. (2017). Rabies Virus Modifies Host Behaviour Through a Snake-Toxin Like Region of Its Glycoprotein That Inhibits Neurotransmitter Receptors in the CNS. Sci. Rep. 7, 1–8. doi: 10.1038/s41598-017-12726-4 28993633 PMC5634495

[B14] Jackson A. C. (2013). History of Rabies Research. USA: Elsevier Science. doi: 10.1016/B978-0-12-396547-9.00001-8

[B15] Lafon M. (2005). Rabies Virus Receptors. J. Neurovirol. 11, 82–87. doi: 10.1080/13550280590900427 15804965

[B16] Laskowski R. A. Jabłońska J. Pravda L. Vařeková R. S. Thornton J. M. (2018). PDBsum: Structural Summaries of PDB Entries. Protein Sci. 27, 129–134. doi: 10.1002/pro.3289 28875543 PMC5734310

[B17] Letunic I. Doerks T. Bork P. (2015). SMART: Recent Updates, New Developments and Status in 2015. Nucleic Acids Res. 43, D257–D260. doi: 10.1093/nar/gku949 25300481 PMC4384020

[B18] Maginnis M. S. (2018). Virus–Receptor Interactions: The Key to Cellular Invasion. J. Mol. Biol. 430, 2590–2611. doi: 10.1016/j.jmb.2018.06.024 29924965 PMC6083867

[B19] Maillard A. P. Gaudin Y. (2002). Rabies Virus Glycoprotein Can Fold in Two Alternative, Antigenically Distinct Conformations Depending on Membrane-Anchor Type. J. Gen. Virol. 83, 1465–1476. doi: 10.1099/0022-1317-83-6-1465 12029162

[B20] Marchler-Bauer A. Derbyshire M. K. Gonzales N. R. Lu S. Chitsaz F. Geer L. Y.. (2015). CDD: Ncbi’s Conserved Domain Database. Nucleic Acids Res. 43, D222–D226. doi: 10.1093/nar/gku1221 25414356 PMC4383992

[B21] Mitchell A. Chang H. Y. Daugherty L. Fraser M. Hunter S. Lopez R.. (2015). The InterPro Protein Families Database: The Classification Resource After 15 Years. Nucleic Acids Res. 43, D213–D221. doi: 10.1093/nar/gku1243 25428371 PMC4383996

[B22] Nilofer C. Sukhwal A. Mohanapriya A. Kangueane P. (2017). Protein-Protein Interfaces are vdW Dominant With Selective H-Bonds and (or) Electrostatics Towards Broad Functional Specificity. Bioinformation 13, 164–173. doi: 10.6026/97320630013164 28729757 PMC5512853

[B23] Omasits U. Ahrens C. H. Müller S. Wollscheid B. (2014). Protter: Interactive Protein Feature Visualization and Integration With Experimental Proteomic Data. Bioinformatics 30, 884–886. doi: 10.1093/bioinformatics/btt607 24162465

[B24] Pantsar T. Poso A. (2018). Binding Affinity via Docking: Fact and Fiction. Molecules 23 (8), 1899. doi: 10.3390/molecules23081899 PMC6222344 30061498

[B25] Pons C. Grosdidier S. Solernou A. Pérez-Cano L. Fernández-Recio J. (2010). Present and Future Chanllenges and Limitations in Protein-Protein Clocking. Proteins Struct. Funct. Bioinforma. 78, 95–108. doi: 10.1002/prot.22564 19731373

[B26] Pulmanausahakul R. Li J. Schnell M. J. Dietzschold B. (2008). The Glycoprotein and the Matrix Protein of Rabies Virus Affect Pathogenicity by Regulating Viral Replication and Facilitating Cell-To-Cell Spread. J. Virol. 82, 2330–2338. doi: 10.1128/jvi.02327-07 18094173 PMC2258906

[B27] Shental-Bechor D. Levy Y. (2008). Effect of Glycosylation on Protein Folding: A Close Look at Thermodynamic Stabilization. Proc. Natl. Acad. Sci. U. S. A. 105, 8256–8261. doi: 10.1073/pnas.0801340105 18550810 PMC2448824

[B28] Shuai L. Wang J. Zhao D. Wen Z. Ge J. He X.. (2019). Integrin β1 Promotes Peripheral Entry by Rabies Virus. J. Virol. 94:e01819–19. doi: 10.1128/jvi.01819-19 PMC6955245 31666383

[B29] Szilagyi A. Zhang Y. (2014). Template-Based Structure Modeling of Protein-Protein Interactions. Curr. Opin. Struct. Biol. 24, 10–23. doi: 10.1016/j.sbi.2013.11.005.Template-based 24721449 PMC3984454

[B30] Tovchigrechko A. Vakser I. A. (2006). GRAMM-X Public Web Server for Protein-Protein Docking. Nucleic Acids Res. 34, 310–314. doi: 10.1093/nar/gkl206 PMC1538913 16845016

[B31] Tovchigrechko A. Wells C. A. Vakser I. A. (2002). Docking of Protein Models. Protein Sci. 11, 1888–1896. doi: 10.1110/ps.4730102 12142443 PMC2373684

[B32] Wang J. Wang Z. Liu R. Shuai L. Wang X. Luo J.. (2018). Metabotropic Glutamate Receptor Subtype 2 Is a Cellular Receptor for Rabies Virus. PloS Pathog. 14, 1–21. doi: 10.1371/journal.ppat.1007189 PMC6070288 30028877

[B33] Wojczyk B. S. Takahashi N. Levy M. T. Andrews D. W. Abrams W. R. Wunner W. H.. (2005). N-Glycosylation at One Rabies Virus Glycoprotein Sequon Influences N-Glycan Processing at a Distant Sequon on the Same Molecule. Glycobiology 15, 655–666. doi: 10.1093/glycob/cwi046 15677380

[B34] Xu H. Hao X. Wang S. Wang Z. Cai M. Jiang J.. (2015). Real-Time Imaging of Rabies Virus Entry Into Living Vero Cells. Sci. Rep. 5, 1–12. doi: 10.1038/srep11753 PMC4493577 26148807

[B35] Yang J. Zhang Y. Rohini K. Srikumar P. S. Anbarasu K. Structural L.. (2010). I-TASSER: A Unified Platform for Automated Protein Structure and Function Prediction. Nat. Protoc. 5, 725–738. doi: 10.1038/nprot.2010.5.I-TASSER 20360767 PMC2849174

