# Supplementary figures and images for: Structural and Evolutionary Insights Into the Binding of Host Receptors by the Rabies Virus Glycoprotein
Source: Front Cell Infect Microbiol. 2021 Oct 11;11:736114. doi: 10.3389/fcimb.2021.736114 (PMC8542875; doi:10.3389/fcimb.2021.736114)

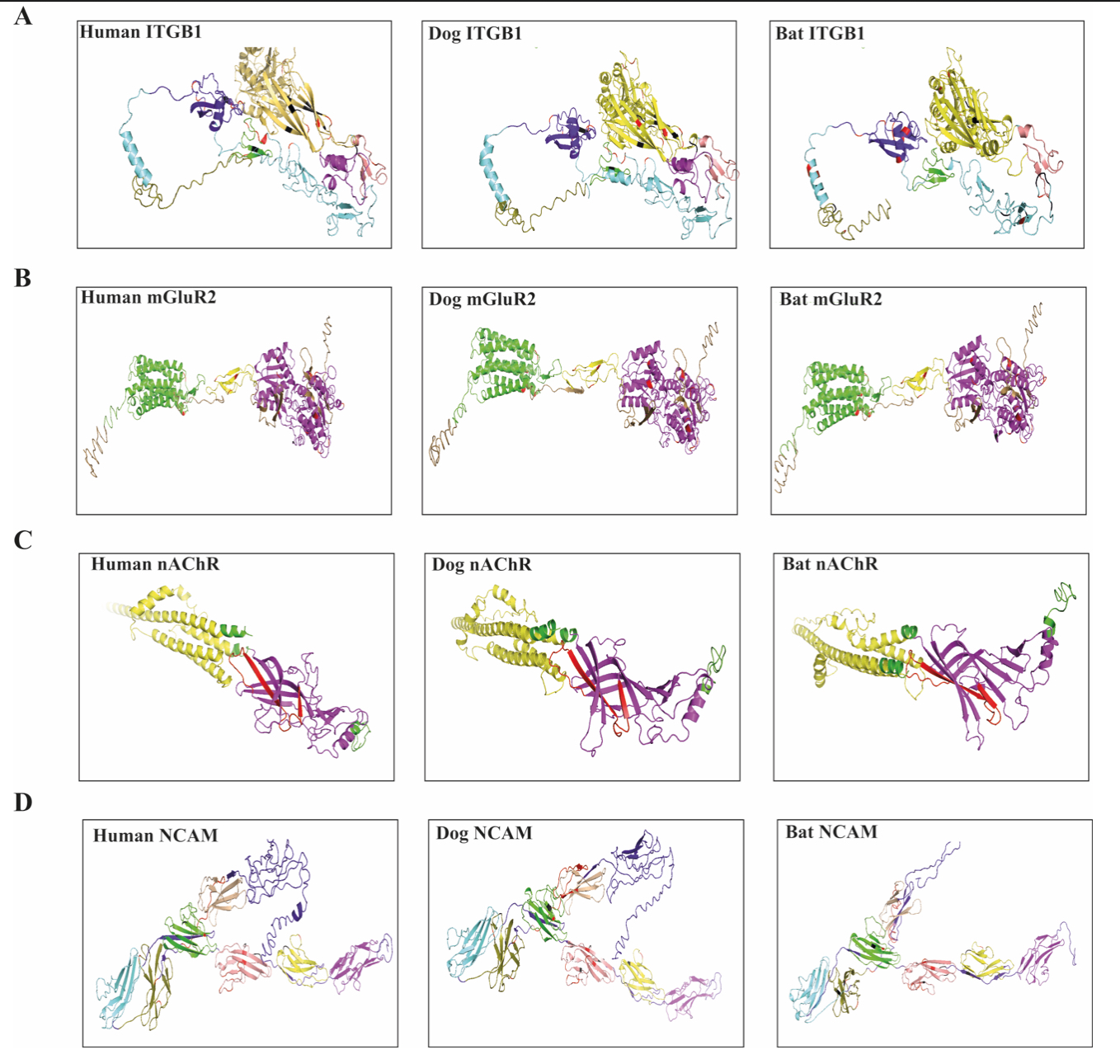

Supplement: Supplementary file 1 [file Image_1.jpg]
